# Epi-aortic lesions, pathologic FMD, endothelial activation and inflammatory markers in advanced naïve HIV-infected patients starting ART therapy

**DOI:** 10.7448/IAS.17.4.19545

**Published:** 2014-11-02

**Authors:** Chiara Bellacosa, Paolo Maggi, Anna Volpe, Sergio Altizio, Nicoletta Ladisa, Silvia Cicalini, Rosaria Viglietti, Antonio Chirianni, Lara Bellazzi, Domenico Zanaboni, Renato Maserati, Canio Martinelli, Paola Corsi, Silvia Sofia, Maurizio Celesia, Ferdinando Sozio, Nicola Abbrescia, Gioacchino Angarano

**Affiliations:** 1 Clinical Infectious Diseases, University of Bari, Policlinico Consorziale, Bari, Italy; 2 INMI L. Spallanzani, IRCCS Roma, Roma, Italy; 3 Clinical Infectious Diseases, Ospedale Cotugno Napoli, Napoli, Italy; 4 Clinical Infectious Diseases, Policlinico San Matteo, Pavia, Italy; 5 Clinical Infectious Diseases, Ospedale Careggi, Firenze, Italy; 6 Clinical Infectious Diseases, Ospedale Garibaldi-Nesima, Catania, Italy; 7 Clinical Infectious Diseases, Ospedale Civile Spirito Santo, Pescara, Italy; 8 IV Clinical Infectious Diseases, Ospedale Cotugno, Napoli, Italy

## Abstract

**Introduction:**

PREVALEAT II (PREmature VAscular LEsions and Antiretroviral Therapy II) is an ongoing multicenter, longitudinal cohort study aimed to the evaluation of cardiovascular (CV) risk in advanced HIV-infected antiretroviral (ARV) naïve patients starting their first antiretroviral therapy (ART).

**Patients and methods:**

All consecutive naïve patients with CD4 cell count<200/mL starting any PI/r-based or NNRTI-based + 2 NRTIs regimen from January 2010 to January 2013 in the participant centres were enrolled. At baseline and after 3 (T1), 6 (T2) and 12 (T3) months patients were subjected to epi-aortic vessels ultrasonography and brachial artery flow mediated dilation (FMD). Viral load, CD4+ cell count, serum lipid values, serum glucose, endothelial activation (ICAM-1 and VCAM-1) and inflammatory markers (IL-6 and hsCRP) values were recorded at the same time. Data about independent risk factors for HIV infection and CV disease are taken at time 0. We enrolled 94 patients: 81% males, 87% caucasians, 40% smokers, 8.2% HCV co-infected and 3.5% with lipodystrophy; 33% of them were homosexuals, 12% drug addicts; 23% were AIDS at presentation. Statistical data analysis has been conducted by the χ^2^ nonparametric method.

**Results:**

In Table 1 it is reported the percentage of patients with pathologic values, moreover, at T3, 60.46% showed undetectable viraemia and 69.77% had CD4 + > 200.

**Conclusions:**

Our data evidence at baseline has a relevant deterioration of CV conditions in terms of ultrasonographic data, FMD, inflammation and cytokine markers among advanced naïves. During follow-up epi-aortic lesions tend to worsen but not significantly, percentage of pathologic FMD remains stable. Regarding markers of endothelial activation ICAM-1 significantly worsens during the period of observation; also VCAM-1 has a trend towards the worsening while not significantly. Conversely, a significant improvement was observed for the markers of inflammation D-dimers and high sensitivity C-reactive protein (hsCRP). IL-6 improved but not significantly. Serum lipid profile shows an increase of HDLc and total cholesterol, but not of LDLc. In conclusion, after a twelve-month follow-up period, CV risk of the patients remains high. ARV therapy seems in fact to improve only non-specific and poor sensitive inflammation biomarkers and HDLc; markers of endothelial activations tend to worsen, intima-media ultrasonography and FMD do not show relevant modifications. Further data are warranted to better understand the role of the different ARV regimens.

**Table 1:** Percentage of patients with pathologic values

	Baseline	T1	T1	T2	T2	T3	T3
	
	%	%	p	%	p	%	p
EPI-aortic lesions	48.78	36.78	0.93	48.80	0.34	62.79	0.42
Pathologic FMD	34.54	38.89	0.78	31.43	0.82	26.09	0.59
ICAM-1	75.32	72.89	0.27	85.10	0.1943	92.86	0.018
VCAM-1	85.71	78.44	0.26	87.23	0.8113	95.24	0.111
IL-6	37.66	50	0.16	40.42	0.75	21.43	0.06
D-dimers	55.84	26.7	0.0009*	19.15	<0.0001*	16.67	<0.0001*
HS-CRP	37.66	22.22	0.116	19.15	0.09	16.67	0.017*
Total cholesterol	9.64	30.43	0.001*	33.82	0.0001*	41.86	<0.00001*
HDLC	71.08	52.1	0.16	51.67	0.18	44.19	0.0032*
LDLC	2.4	7.25	0.16	8.62	0.09	2.33	0.98
Triglycerides	33.73	46.30	0.11	46.03	0.13	37.21	0.71
Glycaemia	18.07	21.74	0.57	7.94	0.08	11.63	0.35

% = percentage of patients; p = statistical values

* = statistically significant.

